# Effect of Pulmonary Hypertension on Survival Outcomes in Patients With Transcatheter Aortic Valve Replacement: A Systematic Review and Meta-Analysis

**DOI:** 10.7759/cureus.58540

**Published:** 2024-04-18

**Authors:** Sulafa Khalil, Godfrey Tabowei, Mandeep Kaur, Samuel K Dadzie, Sajog Kansakar, Merid Moqattash, Praveen Kumar Komminni, Sujith K Palleti

**Affiliations:** 1 Pediatrics, Hamad Medical Corporation, Doha, QAT; 2 Internal Medicine, Texas Tech University Health Sciences Center, Odessa, USA; 3 Internal Medicine, HCA Capital Hospital, Tallahassee, USA; 4 Internal Medicine, Piedmont Athens Regional Medical Center, Athens, USA; 5 Internal Medicine, Maimonides Medical Center, Brooklyn, USA; 6 Medicine, University of Pécs Medical School, Pécs, HUN; 7 Internal Medicine, Suraksha Hospital, Khammam, IND; 8 Nephrology, Louisiana State University Health Sciences Center, Shreveport, USA

**Keywords:** systematic review and meta analysis, cardiovascular mortality, all-cause mortality, transcatheter aortic valve replacement, pulmonary hypertension

## Abstract

The aim of this meta-analysis was to determine the effect of pulmonary hypertension (PH) on survival in patients undergoing transcatheter aortic valve replacement (TAVR). The present study was conducted according to the guidelines of Preferred Reporting of Systematic Review and Meta-Analysis (PRISMA). We conducted a comprehensive search of electronic databases including PubMed/MEDLINE, Embase, Cochrane Library, and Web of Science from January 1, 2015, to March 10, 2024. Outcomes assessed in this meta-analysis included early and late all-cause mortality and cardiovascular mortality. Total 15 studies were integrated into the pooled analysis to assess the impact of PH on outcomes among patients undergoing TAVR, comprising a total sample size of 35,732 individuals. The pooled prevalence of PH stood at 52.57% (n=18,767). Predominantly, the studies were conducted in the United States (n=6), followed by Germany (n=3), with one study each from Japan, Italy, Switzerland, Brazil, Poland, and Australia. Pooled analysis showed that risk of short-term mortality was greater in patients with PH compared to patients without PH (risk ratio (RR): 1.46, 95% CI: 1.19 to 1.80). Risk of long-term mortality was greater in patients with PH (RR: 1.42, 95% CI: 1.29 to 1.55). Risk of cardiovascular mortality was also greater in patients with PH compared to patients without PH (RR: 1.66, 95% CI: 1.36 to 2.02). We advocate for further research to address gaps in understanding different types of PH and their impacts on mortality and cardiovascular outcomes.

## Introduction and background

Pulmonary hypertension (PH) frequently occurs in patients diagnosed with severe aortic stenosis (AS), correlating with heightened mortality rates post-transcatheter aortic valve replacement (TAVR) and surgical aortic valve replacement (SAVR) [1,2]. Some studies indicate PH as an independent risk factor for cardiovascular and overall mortality subsequent to transcatheter aortic valve implantation (TAVI), alongside its well-established association with adverse outcomes following SAVR [3,4]. While TAVI emerges as a safe and efficacious treatment for AS, surpassing SAVR in attention, the heightened perioperative risk posed by PH necessitates thorough investigation into its balance of benefits and risks within this patient cohort [5]. Patients with PH undergoing TAVR may encounter elevated rates of post-procedure complications, prolonged hospital stays, increased mortality, and diminished functional outcomes compared to their counterparts without PH [6]. These findings underscore the importance of meticulous evaluation and management of PH in TAVR patients to optimize outcomes and enhance patient care. Furthermore, patients with PH may face an augmented risk of developing complications, such as sepsis, neurological issues like brain edema and hemorrhagic transformation, bleeding complications including gastrointestinal bleeding, and cardiovascular complications like congestive heart failure and atrial fibrillation with rapid ventricular response [7].

The evidence concerning short- and long-term mortality related to PH following TAVR remains inconclusive. Some studies suggest potential mortality benefits from ameliorating PH post-TAVR, yet a definitive conclusion is lacking. Moreover, the absence of a definitive PH threshold complicates the ability to predict which patients should refrain from TAVR or are at higher risk for post-TAVR mortality and morbidity. For instance, while PH (defined as pulmonary artery systolic pressure (PASP) ≥ 60 mmHg) is considered in the EuroSCORE criteria, it is not integrated into the Society of Thoracic Surgeons score [8]. The disparity in PH definitions employed across studies (e.g., elevated PASP or mean pulmonary artery pressure) further obfuscates the establishment of clear-cut values influencing mortality post-TAVR. Consequently, our objective in this study is to systematically review the literature and quantitatively synthesize data on the relationship between baseline PH and early as well as late cardiovascular and overall mortality.

## Review

Methodology 

Search Strategy 

The present study was conducted according to the guidelines of Preferred Reporting of Systematic Review and Meta-analysis (PRISMA). We conducted a comprehensive search of electronic databases including PubMed/MEDLINE, Embase, Cochrane Library, and Web of Science from January 1, 2015, to March 10, 2024. The search strategy combined keywords and MeSH terms related to ("pulmonary hypertension"[MeSH] OR "PH"[MeSH]) AND ("TAVR"[MeSH] OR "Transcatheter Aortic Valve Replacement"[MeSH]) AND ("death"[MeSH] OR "All-cause mortality"[MeSH] OR "cardiovascular mortality"[MeSH] OR "in-hospital mortality"[MeSH]). Boolean operators (AND, OR) were used to refine the search and ensure inclusivity. Additionally, we manually screened reference lists of relevant articles and reviews for additional studies. Search was performed by two authors independently and disagreements between two authors were resolved through consensus. 

Study Selection 

Two independent reviewers screened titles and abstracts of retrieved studies followed by full-text screening for eligibility based on predetermined inclusion and exclusion criteria. Studies were included if they: (1) involved adult patients (age ≥ 18 years) undergoing TAVR, (2) reported baseline PH status, (3) assessed cardiovascular and overall mortality outcomes, and (4) were written in English. Studies were excluded if they were case reports, reviews, or editorials. Any discrepancies in study selection were resolved through discussion or consultation with a third reviewer. 

Data Extraction and Quality Assessment 

Data extraction was performed independently by two reviewers using a standardized form. Extracted data included study characteristics (e.g., author name, year of publication, study region and sample size), patient demographics, definition of PH, follow-up duration, and outcome measures. Outcomes assessed in this meta-analysis included short term mortality (in-hospital and 30-day mortality), long-term mortality and cardiovascular related mortality. Quality assessment of included studies was performed using New-Castle Ottawa scale. 

Data Analysis 

In this meta-analysis, we employed random-effects models to conduct our analysis, allowing for the calculation of pooled effect estimates, specifically the risk ratio (RR), along with corresponding 95% CI, to evaluate the association between baseline PH and outcomes. We considered a p-value less than 0.05 as indicative of statistical significance. To assess heterogeneity among the included studies, we utilized the I^2^ statistic, where values exceeding 50% were deemed to indicate significant heterogeneity. All statistical analyses were carried out using RevMan software. Forest plots were used to present the pooled findings of included studies.

Results

**Figure 1:**
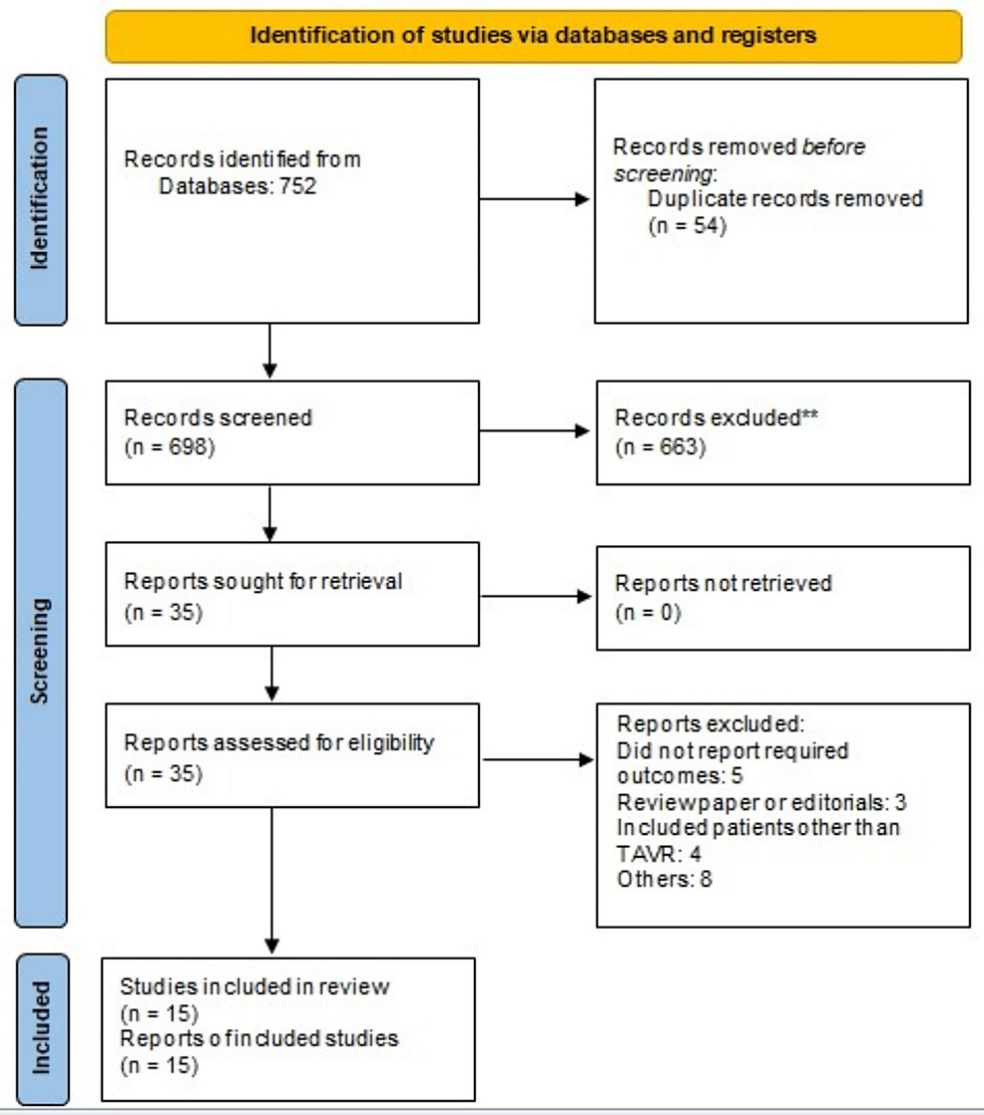
PRISMA flowchart of study selection PRISMA: Preferred Reporting of Systematic Review and Meta-Analysis

Our screening across major databases produced 752 results, which were then refined to 698 articles following the removal of duplicates. Screening occurred in two stages. Initially, abstracts and titles were scrutinized against predetermined inclusion and exclusion criteria. Subsequently, the full texts of eligible studies were retrieved, and a comprehensive evaluation was conducted to determine their suitability for inclusion in this meta-analysis. Ultimately, 15 studies were integrated into the pooled analysis to assess the impact of PH on outcomes among patients undergoing TAVR, comprising a total sample size of 35,732 individuals. Figure 1 illustrates the PRISMA flowchart, delineating the study selection process.

Characteristics of Included Studies

The characteristics of the included studies are presented in Table 1. The pooled sample encompassed 35,732 patients. Among the included studies, the sample sizes varied from 136 to 25,969 patients undergoing TAVR. The pooled prevalence of PH stood at 52.57% (n= 18,767). Predominantly, the studies were conducted in the United States (n=6), followed by Germany (n=3), with one study each from Japan, Italy, Switzerland, Brazil, Poland, and Australia. The majority of the studies utilized mean pulmonary arterial pressure (mPAP) as a diagnostic criterion for PH. Table 2 presents quality assessment of included studies.

**Table 1:** Characteristics of included studies NS: Not specified; PH: Pulmonary hypertension; mPAP: Mean pulmonary arterial pressure; sPAP: Systolic pulmonary arterial pressure

Author	Year	Study Design	Region	Criteria to assess PH	Groups	Sample Size	Follow-up	Age	Males	Previous MI	Diabetes	Afib
Ahmad et al [9]	2023	Retrospective	United States	mPAP≥25 mmHg	Normal	150	30 days	NS	87	NS	40	35
					PH	324			165		155	116
Alushi et al [10]	2019	Retrospective	Germany	sPAP≥34 mmHg	Normal	136	365 days	NS	NS	NS	NS	NS
					PH	481						
Barbash et al [11]	2016	Prospective	United States	sPAP> 50 mmHg	Normal	172	365 days	83	116	48	69	92
					PH	243		84	80	23	55	73
Keymel et al [12]	2020	Retrospective	Germany	mPAP≥25 mmHg	Normal	52	30 days	78	35	10	16	NS
					PH	73		78.9	49	20	28	
Kleczynski et al [13]	2017	Prospective	Poland	NS	Normal	83	365 days	82	28	20	23	29
					PH	65		82	28	28	25	23
Lindman et al [14]	2016	Prospective	United States	mPAP≥35 mmHg	Normal	785	365 days	85	409	168	247	NS
					PH	1395		83	741	382	570	
Masri et al [15]	2018	Retrospective	United States	mPAP≥25 mmHg	Normal	134	700 days	82.9	66	75	48	43
					PH	102		82.8	50	33	38	64
Mayr et al [16]	2021	Retrospective	Germany	NS	Normal	359	30 days	81	225	NS	NS	NS
					PH	718		81	432			
Miyamoto et al [17]	2022	Retrospective	Japan	sPAP>36 mmHg	Normal	1027	700 days	84.5	429	NS	263	229
					PH	845		84.7	141		108	173
Mujeeb et al [18]	2021	Retrospective	United States	NS	Normal	12989	30 days	80.8	6118	NS	4845	7118
					PH	12980		80.9	6243		4893	7126
Naing et al [19]	2022	Prospective	Australia	mPAP>20 mmHg	Normal	320	365 days	NS	NS	NS	NS	NS
					PH	179						
O Sullivan et al [20]	2015	Prospective	Switzerland	mPAP≥25 mmHg	Normal	108	365 days	81.7	60	17	31	8
					PH	325		82.5	136	48	95	52
Souza et al [21]	2015	Prospective	Brazil	NS	Normal	103	365 days	NS	NS	NS	NS	NS
					PH	33						
Sultan et al [22]	2020	Retrospective	United States	mPAP>25 mmHg	Normal	201	1,825 days	82.9	97	58	73	53
					PH	360		82.1	189	146	161	146
Testa et al [23]	2016	Retrospective	Italy	sPAP>40 mmHg	Normal	346	365 days	82	152	72	90	69
					PH	644		78.5	300	133	184	163

**Table 2:** Quality assessment of included studies

Author	Selection	Comparability	Outcome or Exposure Assessment	Overall
Ahmad et al [9]	3	2	3	Good
Alushi et al [10]	3	2	3	Good
Barbash et al [11]	3	2	2	Good
Keymel et al [12]	4	2	3	Good
Kleczynski et al [13]	4	1	3	Good
Lindman et al [14]	3	1	2	Fair
Masri et al [15]	3	2	2	Good
Mayr et al [16]	3	1	3	Good
Miyamoto et al [17]	3	2	2	Good
Mujeeb et al [18]	3	1	3	Good
Naing et al [19]	4	2	3	Good
O Sullivan et al [20]	3	2	3	Good
Souza et al [21]	3	2	2	Good
Sultan et al [22]	4	1	2	Good
Testa et al [23]	3	2	3	Good

Effect of PH on Short-Term Mortality 

**Figure 2:**
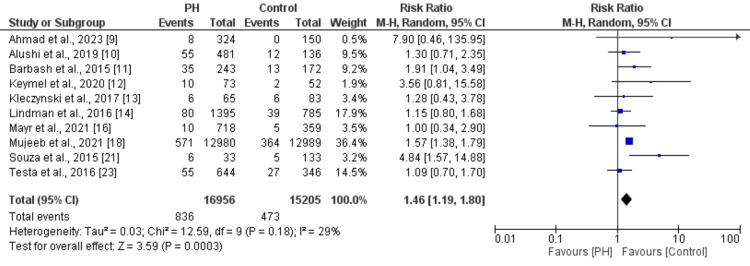
Effect of PH on short-term mortality [9-14,16,18,21,23] PH: Pulmonary hypertension

10 studies were included in the pooled analysis of estimating the effect of PH on short-term mortality in patients undergoing TAVR; the results are presented in Figure 2. Pooled analysis showed that risk of short-term mortality was 1.46 times greater in patients with PH compared to patients without PH (RR: 1.46, 95% CI: 1.19 to 1.80). No significant heterogeneity was reported among the study results (I^2^: 29%). 

Effect of PH on Long-Term Mortality 

**Figure 3:**
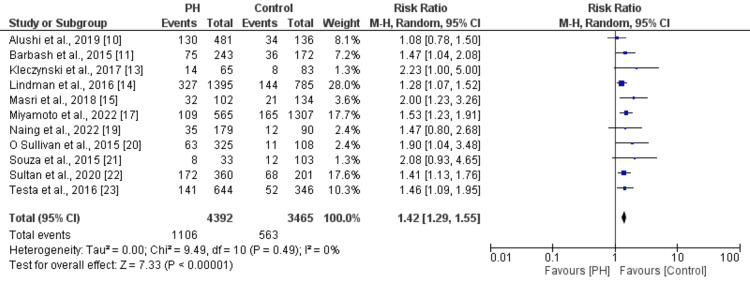
Effect of PH on long-term mortality in patients undergoing TAVR [10-15,17,19-23] PH: Pulmonary hypertension

11 studies were included in the pooled analysis of estimating the effect of PH on long-term mortality in patients undergoing TAVR; the results are presented in Figure 3. Pooled analysis showed that risk of long-term mortality was 1.42 times greater in patients with PH compared to patients without PH (RR: 1.42, 95% CI: 1.29 to 1.55). No significant heterogeneity was reported among the study results (I^2^: 0%). 

Effect of PH on Cardiovascular Mortality 

**Figure 4:**
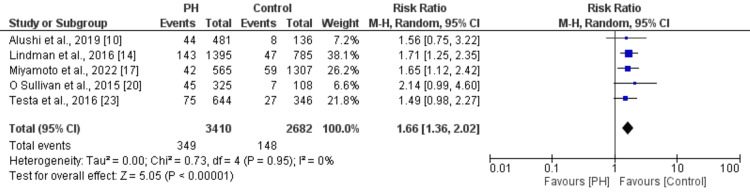
Effect of PH on cardiovascular mortality in patients undergoing TAVR [10,14,17,20,23] PH: Pulmonary hypertension

Five studies were included in the pooled analysis of estimating the effect of PH on cardiovascular mortality in patients undergoing TAVR; the results are presented in Figure 4. Pooled analysis showed that risk of cardiovascular mortality was 1.66 times greater in patients with PH compared to patients without PH (RR: 1.66, 95% CI: 1.36 to 2.02). No significant heterogeneity was reported among the study results (I^2^: 0%). 

Discussion

This meta-analysis, encompassing 15 studies published from 2015, aggregated individual findings concerning three distinct outcomes. Our analysis indicates a significant contrast in short-term mortality, long-term mortality, and cardiovascular mortality, with all these outcomes exhibiting notably higher risks following TAVR in patients with PH compared to those without PH at baseline.

Our findings align with previous studies underscoring the prognostic significance of baseline PH in patients with severe AS undergoing TAVI procedures. Tang et al. [23] conducted a comprehensive meta-analysis on this subject, echoing the impact of baseline PH on post-TAVI mortality. Despite numerous studies investigating the association between PH and post-TAVI outcomes, determining the optimal cutoff point for defining PH as a risk factor in the preoperative assessment of these patients remains unresolved.

Numerous studies have highlighted the association of baseline PH with delayed rather than immediate mortality and poorer clinical outcomes in patients undergoing TAVR [3,24]. Luçon et al found that baseline PH served as a predictive factor for one-year mortality post-TAVR, while Testa et al showed that baseline systolic pulmonary arterial pressure (sPAP) exceeding 60 mmHg independently predicted one-year mortality [2,24]. In line with these findings, our study revealed that baseline PH was linked to worse clinical outcomes at the two-year mark compared to patients without PH. Moreover, higher baseline sPAP levels correlated with increased occurrence of adverse clinical events. A meta-analysis from China and the US echoed the observations of the observational studies included in our analysis [25-26]. The randomized controlled trial by Lindman et al and a retrospective study recommended considering both objective PH findings and clinical factors in assessing mortality risk and guiding decisions regarding TAVR [14,27]. While Kleczynski et al demonstrated elevated all-cause mortality by stratifying PH based solely on tricuspid regurgitation velocity (TRV), they found no impact on quality of life in PH patients; however, further studies with larger cohorts are warranted to validate these findings [13].

An important finding of this study is the significant association between combined PH and heightened mortality risk compared to the absence of PH. However, there was no notable difference observed between precapillary PH, isolated PH, and the absence of PH in terms of all-cause mortality [20,22]. It's worth noting that only two of the included studies directly compared mortality risk across different types of PH. Consequently, we advocate for further research to delve into and compare mortality and cardiovascular risks among the various types of PH. Furthermore, baseline PH (defined as mPAP ≥ 25 mmHg) is recognized as one of the risk factors incorporated in the pulmonary, bleeding, osler, sex, and skeletal complications score (PBOSS) for predicting TAVI outcomes in chronic obstructive pulmonary disorder (COPD) patients, alongside parameters such as BMI (below 21 kg/m^2^), oxygen dependency, and covering less than 200 m in the six-minute walk test. The adverse outcomes observed in patients with baseline PH underscore the necessity for continued investigation and the development of innovative therapies aimed at reducing PASP alongside TAVI for AS [28,29]. Indeed, considering the well-established link between post-TAVI PASP levels and subsequent survival, the imperative for supplementary medical interventions targeting PASP reduction warrants attention in forthcoming studies [30].

Currently, the only established scoring systems that take into account pre-TAVI PH values are the euroSCORE, which incorporates PASP assessed via echocardiography at 60 mmHg and the PBOSS score, designed specifically for risk assessment in COPD patients. Although most studies analyzed in our meta-analysis utilized the mPAP score, they applied different cutoff values. It is crucial for future research efforts to focus on determining mortality rates across different cutoff points to fully understand the implications of PH.

This meta-analysis was conducted using real-world studies, and therefore, our findings should be understood within the framework of observational research and its inherent limitations. Additionally, several of the included observational studies were retrospective in nature, which heightens the potential for bias. There was also a lack of consistency among the studies regarding the definition and diagnostic method of PH, which could lead to underestimation or overestimation of the study results. Furthermore, only two studies directly compared the risk of outcomes among different types of PH. Consequently, future research endeavors should prioritize the comparison of outcomes across various types of PH.

## Conclusions

In conclusion, our meta-analysis of 15 studies on PH and outcomes post-TAVR reveals significant associations with short-term mortality, long-term mortality, and cardiovascular mortality. Despite variations in study designs and definitions of PH, our findings underscore the heightened risks faced by patients with PH undergoing TAVR. We advocate for further research to address gaps in understanding different types of PH and their impacts on mortality and cardiovascular outcomes. Moreover, the need for standardized diagnostic criteria and cutoff points for PH assessment in TAVR patients is evident, emphasizing the importance of ongoing investigation in this area.
